# Gamer's Thrombosis: A Review of Published Reports

**DOI:** 10.31486/toj.19.0058

**Published:** 2020

**Authors:** Kerry Anne Rambaran, Saeed K. Alzghari

**Affiliations:** ^1^ Department of Pharmacy, Scripps Mercy Hospital San Diego, San Diego, CA; ^2^ Department of Pharmacotherapy, University of North Texas Health Science Center College of Pharmacy, Fort Worth, TX; ^3^ Department of Pharmacy Practice, Texas Tech University Health Sciences Center Jerry H. Hodge School of Pharmacy, Dallas, TX

**Keywords:** *Pulmonary embolism*, *thrombosis*, *venous thrombosis*, *video games*

## Abstract

**Background:** Thrombosis, well known as a condition of the elderly, is occurring in the otherwise healthy adolescent population. Immobility is a significant risk factor for venous thromboembolism (VTE), and adolescents who play video games are immobile for extended periods of time. Some are presenting with VTE. When other risk factors such as obesity are present, the risk of VTE formation increases. We provide a review of published case reports regarding gaming and thrombosis.

**Methods:** We searched PubMed, Scopus, Web of Science, and EBSCO for articles published through July 2019, using the keywords “computer game thrombosis,” “computer game pulmonary embolism,” “computer game deep vein thrombosis,” “video game thrombosis,” “video game pulmonary embolism,” and “video game deep vein thrombosis.”

**Results:** Of the 26 articles we identified, we included 12 articles in our review that report a total of 15 cases, of which 2 resulted in fatalities. Modifiable risk factors included cigarette use, being overweight, birth control use, and prolonged immobility. Anticoagulation was the principal treatment modality in patients presenting with gaming thrombosis.

**Conclusion:** We strongly encourage screening gamers for possible VTEs if clinically warranted.

## INTRODUCTION

Venous thromboembolism (VTE), a blood clot that starts in a vein, typically has 2 types: deep vein thrombosis (DVT), which occurs when the clot occurs in a deep vein such as the leg, and pulmonary embolism (PE), which occurs when a DVT breaks off and enters the arteries of the lungs. Approximately 300,000 to 600,000 cases of VTE occur annually in the United States, approximately 33% of patients will have a recurrence within 10 years, and 10% to 30% of patients die within 1 month of diagnosis.^1^ Not only is VTE associated with considerable morbidity and mortality, but VTE cases also account for a high financial burden in healthcare, with an annual estimated cost of $2 to $10 billion.^1^ Historically, VTE has been known principally as a condition of the elderly; however, this definition is changing to include the otherwise healthy population of adolescents. A retrospective study of cases from the Pediatric Health Information System administrative database showed that from 2001 to 2007, adolescents accounted for 30% of admissions for VTE, and other articles report similar findings across the pediatric and adolescent population.^2-6^

Numerous risk factors, both reversible and irreversible, can foster a hypercoagulable state and thus predispose patients to the development of a VTE. The Virchow triad illustrates 3 of the important factors—hemostasis, hypercoagulability, and vessel wall injury—that alter blood flow and, in turn, cause or facilitate thrombus formation.^7^ The most common reversible factors in both adolescents and adults are obesity and sedentariness.^8-10^ Prolonged immobility in adolescents is often attributable to playing video games. In this review, we summarize the reported cases related to thrombosis in gamers and describe the clinical implications.

## METHODS

We searched PubMed, Scopus, Web of Science, and EBSCO for articles published through July 2019, using the keywords “computer game thrombosis,” “computer game pulmonary embolism,” “computer game deep vein thrombosis,” “video game thrombosis,” “video game pulmonary embolism,” and “video game deep vein thrombosis.” To be included in this review, articles had to meet the following inclusion criteria: (1) must be full-text articles; (2) must involve humans; (3) must be a randomized controlled trial, prospective trial, retrospective analysis, case series, or case report; and (4) must include clinical findings at presentation. The senior author (SKA) identified relevant articles reporting the outcomes of interest. The lead author (KAR) extracted data from all reports, and SKA resolved any discrepancies. We checked the reference lists of each relevant article for additional resources that met the selection criteria.

## RESULTS

We initially identified 26 articles; after applying the inclusion criteria, 12 relevant articles were selected for this review (Figure), representing 15 patients who ranged in age from 12 to 44 years.^11-22^ Among these 15 patients, recurrent VTE occurred in 3 patients, and 2 patients died as a result of VTE.^12,14,20,21^ One of the cases was reported postmortem, so an approximate duration of gaming is provided. Five patients lived in the United States, 3 in New Zealand, 3 in Singapore, 2 in Korea, and 1 each in Austria and the United Kingdom. Three patients had DVT, 5 had PE, and 7 had both DVT and PE. Concerning risk factors, 2 patients used cigarettes, 2 patients had factor V Leiden (FVL) heterozygosity, 5 patients had a sedentary lifestyle, 2 patients had a family history of VTE, 1 patient had May-Thurner syndrome, 1 patient had Klinefelter syndrome, 1 patient was taking birth control, 1 patient had Guillain-Barré syndrome (GBS), and 1 patient had prior bariatric surgery. Seven of the 15 patient cases reported body mass index (BMI) data, and all 7 patients had a BMI >25 kg/m^2^. Twelve of the 15 patients received anticoagulation treatment (Table).

**Figure .**
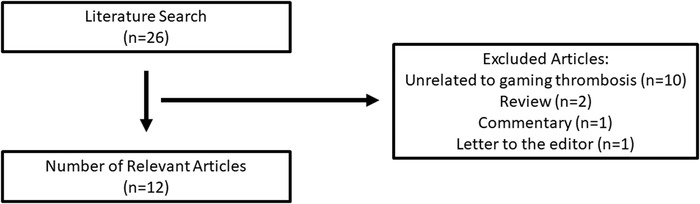
**Article search and selection diagram.**

**Table.** Cases of Gaming Thrombosis

Case	Sex, Age	Thrombosis Type	Gaming Time	BMI, kg/m^2^	Cigarette Use	FVL Hetero-zygosity	Sedentary Lifestyle	Family History	Other Risk Factors	Treatment	Death	Recurrence
Braithwaite et al, 2018^11^	M, 44	PE	36 h	48.4	Yes	No	Yes	No	Bariatric surgery	6 months of warfarin with LMWH bridge; aspirin; LMC	No	No
Kohorst et al, 2018^12^	M, 18	PE	Up to 12 h/d	37	No	Yes	No	No	No	6 months of AC; LMC	No	No
Kohorst et al, 2018^12^	M, 15	PE, DVT	4-12 h/d	50	No	No	Yes	No	No	6 months of AC; weight management program	No	No
Kohorst et al, 2018^12^	M, 13	PE, DVT	Several h/d	56	No	No	Yes	Yes	GBS	3 months of AC; 6 months of PT	No	No
Kohorst et al, 2018^12^	M, 17	PE, DVT	Several h	39	No	Yes	Yes	No	MTS	6 months of AC; LMC	No	Yes
Brodmann et al, 2015^13^	F, 21	PE, DVT	3 h	N/A	No	No	No	Yes	Second- generation birth control pills	Surgery for thrombus removal with UFH during the procedure; IVC filter	No	No
Lee et al, 2015^14^	M, 16	PE, DVT	>13 h/d	N/A	No	No	Yes	No		2.5 years of AC; IVC filter	Yes	Yes
Braithwaite et al, 2014^15^	M, 42	PE	48 h	38	No	No	No	No	Klinefelter syndrome	6 months of warfarin with LMWH bridge; LMC	No	No
Chang et al, 2013^16^	M, 31	DVT	7-8 h/d	N/A	No	No	No	No	No	Thrombolysis with UFH and urokinase	No	No
Hayes et al, 2013^17^	M, 15	PE	16-18 h	N/A	No	No	No	No	No	PTE with UFH and tPa; warfarin therapy	No	No
Kim et al, 2009^18^	M, 36	PE, DVT	12 h/d for 2 weeks	29.4	Yes	No	No	No	No	Thrombolysis with UFH and tPa; 6 months of warfarin with LMWH bridge	No	No
Phipps and Ng, 2008^19^	M, 33	DVT	2-3 h/d	N/A	No	No	No	No	No	AC; LMC	No	No
Chew, 2006^20^	M, 16	PE, DVT	3 h	N/A	No	No	No	No	No	Thrombolysis; IVC filter; warfarin therapy	No	Yes
Lee, 2004^21^	M, 24	PE	80 h	N/A	No	No	No	No	No	Found deceased in bathroom	Yes	No
Ng et al, 2003^22^	M, 12	DVT	4 h	N/A	No	No	No	No	No	AC	No	No

AC, anticoagulation; BMI, body mass index; DVT, deep vein thrombosis; F, female; FVL, factor V Leiden; GBS, Guillain-Barré syndrome; IVC, inferior vena cava; LMC, lifestyle modification counseling; LMWH, low molecular weight heparin; M, male; MTS, May-Thurner syndrome; N/A, not available; PE, pulmonary embolism; PTE, pulmonary thromboendarterectomy; tPa, tissue plasminogen activator; UFH, unfractionated heparin; VTE, venous thromboembolism.

## DISCUSSION

More than half of the patients identified in these case reports were adolescents ≤18 years of age (8/15, 53%). Many individuals—young and old—spend hours playing video games online and offline.^23,24^ The prevalence of excessive gaming has been well documented in the literature, showing that, on average, individuals spend more than 10 hours gaming during a session.^25,26^ However, no exact definition of excessive gaming exists. Further, while these cases involved patients who reported excessive gaming, the cause of VTE is multifactorial, and thus the risk of developing a VTE is greater when more risk factors are present.

Lee reported the first death from gaming thrombosis in 2004, and the second death was reported in 2015.^14,21^ Of the 15 cases included in this review, the number of hours a patient spent gaming in a single setting ranged from 3 to 80 hours. Additionally, 9 of the 15 patients had modifiable risk factors including cigarette use, being overweight, birth control use, and prolonged immobility.^11-15,18,21^ One patient was taking a second-generation oral contraceptive; individuals taking oral contraceptive pills have a 2 to 4 times greater risk of VTE than those who do not use this contraceptive method.^9,27,28^

Goldhaber and colleagues investigated the risk factors for PE in women and reported that obesity, cigarette smoking, and hypertension were the major contributors.^29^ The Ageno et al metaanalysis assessing the association between cardiovascular risk factors and VTE found that “compared with control subjects, the risk of VTE was 2.33 for obesity (95% confidence interval [CI], 1.68 to 3.24), 1.51 for hypertension (95% CI, 1.23 to 1.85), 1.42 for diabetes mellitus (95% CI, 1.12 to 1.77), 1.18 for smoking (95% CI, 0.95 to 1.46), and 1.16 for hypercholesterolemia (95% CI, 0.67 to 2.02).”^30^ The authors concluded that cardiovascular risk factors are associated with VTE. The Tromsø study showed that heavy smokers (>20 pack-years) had an increased risk for total VTE (hazard ratio [HR] 1.46) and provoked VTE (HR 1.75) compared to never smokers.^31^ Similarly, the Multiple Environmental and Genetic Assessment of risk factors for VTE (MEGA study) reported that overweight and obese individuals were associated with a 1.7- and 2.4-fold increase in VTE, respectively, compared to individuals of healthy weight.^32^

Many of the patients included in this review presented with risk factors identified in these studies. Thus, educating and counseling patients on lifestyle modification to prevent cardiovascular and VTE complications is important. Of note, 5 patients received lifestyle management counseling, 1 was enrolled in a weight management program, and 1 received physical therapy after DVT and PE occurrence.^11,12,15,19^

Several patients included in this review had nonmodifiable risk factors. As previously noted, 2 patients had FVL heterozygosity, an abnormal version of factor V that is not affected by activated protein C (APC). Because FVL is not affected by APC, clot formation is prolonged, and these patients would seem to have a greater risk for thrombosis; however, the evidence is conflicting as to whether the presence of FVL heterozygosity increases risk for VTE.^33,34^

One patient presented with GBS, a condition in which the body's immune system attacks peripheral nerves, potentially leading to muscle weakness or loss of sensation in the extremities. This patient had 3 weeks of immobility secondary to GBS and had a sedentary lifestyle at baseline. A retrospective study of 73 patients admitted to a hospital for GBS showed that 5 patients had a DVT postdischarge, and 4 were anticoagulated with prophylactic low molecular weight heparin; the authors concluded that full anticoagulation may be needed for patients with GBS.^35^

One patient presented with May-Thurner syndrome in which the left iliac artery is compressed by the right iliac artery, thus predisposing the patient to left extremity DVT. This condition is generally not life threatening, as >70% compression is needed to result in a DVT.^36^ However, a clot that breaks off and travels to the lungs can become life threatening, which is possibly what occurred in this case because this patient presented with a PE.

One patient presented with Klinefelter syndrome which is associated with VTE and is comparable to inherited thrombophilias. The mechanism behind the association between Klinefelter syndrome and VTE is multifactorial, and the current literature on this topic is limited.^37-39^

For treatment of VTE, the Antithrombotic Therapy for VTE Disease: CHEST Guideline and Expert Panel Report states that adults should receive anticoagulation for 3 months, and if a patient has a low or moderate risk of bleeding, anticoagulation should be continued without a scheduled stop date.^40^ According to the CHEST guideline, direct oral anticoagulants are preferred to vitamin K antagonists; in turn, vitamin K antagonists are preferred to low molecular weight heparin. For treatment of pediatric patients, the American Society of Hematology (ASH) guidelines suggest 6 to 12 months of anticoagulation therapy with either a vitamin K antagonist or low molecular weight heparin in patients with unprovoked VTE.^41^

For the patients included in this review, the duration of therapy varied and was not always consistent with guideline recommendations. Of the 15 cases, 4 adults received 6 months of anticoagulation, and 4 pediatric patients received 3 months to 2.5 years of anticoagulation. The remaining cases did not specify the duration of therapy. Six patients received heparin preparations either secondary to surgery for removal of the clot or as bridging therapy to warfarin. One patient was prescribed aspirin after a 6-month course of warfarin.^11^

In terms of prevention, lifestyle management, such as interrupting the duration of prolonged gaming by walking around every few hours and changing positions, can help prevent a VTE. Such preventive measures are especially important if a patient has risk factors such as being overweight or obese. Patients should quit smoking to reduce their chances of developing VTE.

This clinical review is based on case reports that are not without limitations. Consequently, this information should be interpreted with caution because the majority of the cases, albeit a small number, were reported from countries outside of the United States and therefore may not generalize to the US population.

## CONCLUSION

When an adolescent presents with a VTE, asking the patient or the patient's parents about any history of gaming and the number of hours spent gaming is an important aspect of the workup. Clinicians should follow the current CHEST or ASH guidelines to treat the VTE because specific gaming thrombosis guidelines are not available. Because gaming is prevalent, it should be recognized as a public health issue and a health burden. Well-defined diagnostic guidelines and criteria for gaming disorder are needed to establish appropriate treatment.

## Notes

This article meets the Accreditation Council for Graduate Medical Education and the American Board of Medical Specialties Maintenance of Certification competencies for Patient Care and Medical Knowledge.
